# The Different Faces of Rolling-Circle Replication and Its Multifunctional Initiator Proteins

**DOI:** 10.3389/fmicb.2017.02353

**Published:** 2017-11-30

**Authors:** Paweł Wawrzyniak, Grażyna Płucienniczak, Dariusz Bartosik

**Affiliations:** ^1^Department of Bioengineering, Institute of Biotechnology and Antibiotics, Warsaw, Poland; ^2^Department of Bacterial Genetics, Institute of Microbiology, Faculty of Biology, University of Warsaw, Warsaw, Poland

**Keywords:** rolling-circle replication, transposition, conjugal transfer, multifunctional protein, mobile genetic elements, horizontal gene transfer

## Abstract

Horizontal gene transfer (HGT) contributes greatly to the plasticity and evolution of prokaryotic and eukaryotic genomes. The main carriers of foreign DNA in HGT are mobile genetic elements (MGEs) that have extremely diverse genetic structures and properties. Various strategies are used for the maintenance and spread of MGEs, including (i) vegetative replication, (ii) transposition (and other types of recombination), and (iii) conjugal transfer. In many MGEs, all of these processes are dependent on rolling-circle replication (RCR). RCR is one of the most well characterized models of DNA replication. Although many studies have focused on describing its mechanism, the role of replication initiator proteins has only recently been subject to in-depth analysis, which indicates their involvement in multiple biological process associated with RCR. In this review, we present a general overview of RCR and its impact in HGT. We focus on the molecular characteristics of RCR initiator proteins belonging to the HUH and Rep_*trans* protein families. Despite analogous mechanisms of action these are distinct groups of proteins with different catalytic domain structures. This is the first review describing the multifunctional character of various types of RCR initiator proteins, including the latest discoveries in the field. Recent reports provide evidence that (i) proteins initiating vegetative replication (Rep) or mobilization for conjugal transfer (Mob) may also have integrase (Int) activity, (ii) some Mob proteins are capable of initiating vegetative replication (Rep activity), and (iii) some Rep proteins can act like Mob proteins to mobilize plasmid DNA for conjugal transfer. These findings have significant consequences for our understanding of the role of RCR, not only in DNA metabolism but also in the biology of many MGEs.

## Introduction

**FIGURE 1:**
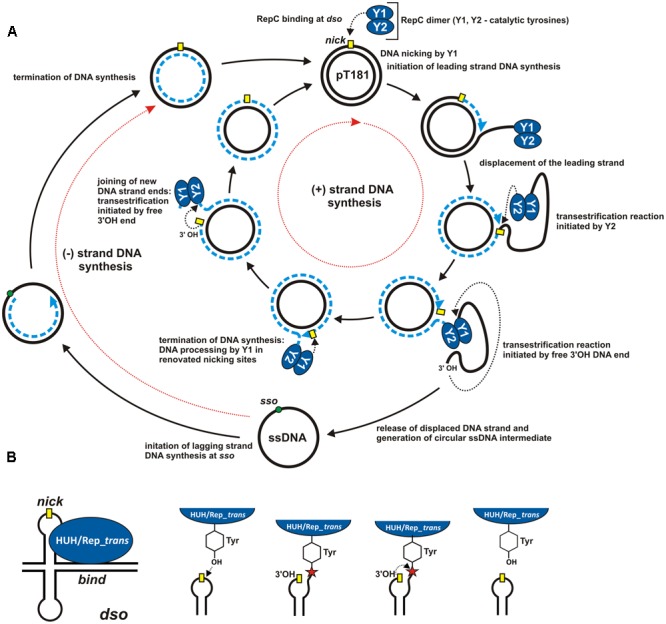
Mechanism of rolling-circle replication. **(A)** A model for RCR based on studies of plasmid pT181. Separated cycles of leading (+) and lagging (–) DNA strand synthesis (initiated at the *dso* and *sso* origins, respectively) are marked by red dotted arrows. The individual steps in the synthesis are described in the Figure and details are given in the text. The RCR initiation protein (RepC) initiates (+) strand replication at the nick site (marked by a yellow rectangle). Dashed blue lines indicate the nascent DNA strand and blue arrows show the direction of synthesis. Black dotted arrows indicate nucleophilic attack of the catalytic tyrosine residue on the phosphodiester bond in the DNA or of the free 3′-OH DNA end on the phosphotyrosine bond between the enzyme and the DNA. **(B)** ssDNA breakage and joining reactions catalyzed by HUH and Rep_*trans* endonucleases at the *dso*. The *nick* site exposed in a hairpin-like structure is shown by a yellow rectangle. The red star indicates the phosphotyrosine bond between the enzyme and the 5′-end of the DNA (see text for details). The reaction catalyzed by HUH/Rep_*trans* is reversible since the released 3′-OH DNA end could acts as a nucleophile on the phosphotyrosine bond (adopted from Chandler et al., 2013; Pastrana et al., 2016).

Rolling-circle replication (RCR) was first described nearly half a century ago following analysis of the replication of *Escherichia coli* bacteriophage ΦX174 (Gilbert and Dressler, 1968; Dressler, 1970). Since then, many other genetic elements replicating by the rolling-circle (RC) mechanism have been identified, including bacteriophages, plasmids of Gram-positive and Gram-negative bacteria, archaeal plasmids and eukaryotic viruses (Baas, 1985; Koonin and Ilyina, 1992; Ruiz-Masó et al., 2015). A specific feature of RCR is its asymmetric character manifested by the separation of leading (+) and lagging (-) DNA strand synthesis. Replication of the (+) and (-) strands is initiated at different replication *origins*. The RCR initiation protein plays a key role in (+) strand replication. It initiates DNA replication by cleaving the leading strand within the corresponding *origin* (**Figure 1A**).

Interestingly, RCR was also found to play an important role in horizontal gene transfer (HGT). For example, it is the initial step in the conjugal transfer of plasmids and integrative and conjugative elements (ICEs) (Llosa et al., 2002), as well as the basis of T-DNA transfer from *Agrobacterium tumefaciens* to plant cells (Zupan and Zambryski, 1995). It is also thought that some transposable elements, such as bacterial IS*91*-like insertion sequences and eukaryotic helitrons, use RCR to replicate in host genomes (Mendiola et al., 1994; Thomas and Pritham, 2015). According to their biological roles, RCR initiator proteins are divided into three groups: Rep proteins (vegetative replication), Mob proteins (mobilization for conjugal transfer) and Tnp proteins (transposition).

A number of reports concerning RCR replication have recently been published (e.g., Wang et al., 2013; Boer et al., 2016; Carr et al., 2016; Wright and Grossman, 2016). These have revealed new facts about RCR and especially the role of the replication initiator proteins. In this review, different RCR variants are compared, and features and functions of selected multifunctional RCR initiator proteins encoded by mobile genetic elements (MGEs) are described.

## RCR Involvement In Various Biological Processes

### RC Mechanism in Vegetative DNA Replication

The best known RCR replicons are bacteriophages and bacterial plasmids, including the aforementioned prototype *E. coli* phage ΦX174 (Henry and Knippers, 1974; Ikeda et al., 1976; Eisenberg and Kornberg, 1979) and the plasmid pT181 isolated from *Staphylococcus aureus* (Koepsel et al., 1985). In both replicons the replication initiator protein interacts with two DNA regions, called the *bind* site and the *nick* site, located within the double-stranded *origin* (*dso*) (**Figure 1B**). The initiator protein interacts with the *bind* locus in a non-covalent manner. The exposed *nick* site of the *dso* is cleaved by the catalytic tyrosine of the initiator protein (**Figure 1B**). The released 3′-OH end then acts as the primer for synthesis of the new DNA strand. The initiator protein remains covalently attached (through a phosphotyrosine bond) to the 5′ end of the nicked strand until leading strand replication is terminated, and the circular ssDNA intermediate is released from the newly replicated dsDNA molecule (**Figure 1A**).

Conversion of ssDNA into dsDNA is initiated at the lagging strand replication *origin*, referred to as the single-strand initiation (*ssi*) sequence in ΦX174 (Masai et al., 1990) or the single-strand *origin* (*sso*) in pT181 (Birch and Khan, 1992). The Rep protein is not involved in this stage of DNA replication; only the enzymatic machinery of the host is required. In ΦX174, primase complex is recruited within the *ssi* and is responsible for the synthesis of the RNA primers. Starting from the *ssi*, primase then moves along the DNA strand, periodically synthesizing additional RNA primers. In pT181, only a single RNA primer is synthesized at the *sso* site by the RNA polymerase.

The RCR mechanisms of ΦX174 and pT181 differ significantly at the leading strand termination stage. This is a consequence of the different life strategies of bacteriophages and bacterial plasmids. In phage ΦX174, termination of leading (+) strand replication initiates another round of replication, enabling the phage to rapidly amplify its genome before lysis and escape from the bacterial cell (Novick, 1998). In contrast, the number of pT181 copies per bacterial cell is tightly regulated. Therefore, after a single replication cycle, RepC, the replication initiator protein of pT181, is inactivated and cannot initiate another round of replication (**Figure 1A**).

Although the crystallographic structures of Protein A of ΦX174 and RepC of pT181 have yet to be determined, detailed molecular analyses have helped to describe their role in the initiation of replication (**Figure 1**). The Rep proteins exhibit nucleolytic activity and they cleave the leading DNA strand to initiate the replication process. The active sites of both these enzymes contain conserved tyrosine residues that are directly involved in the catalytic functions. Protein A has two tyrosines, Y1 and Y2, which are responsible for the initiation and termination of replication, respectively (Mansfeld et al., 1986) (**Figure 1A**). In the active site of RepC of pT181 there is only one tyrosine, but as this Rep protein acts as a dimer, the tyrosine residue of one subunit (Y1) initiates replication, while that of the other (Y2) terminates it (Rasooly and Novick, 1993) (**Figure 1A**). These two structural arrangements seems to be preferred since prokaryotic RCR initiator proteins are mainly purified as a monomers or dimers (Ruiz-Masó et al., 2015).

Interestingly, the RepB protein of the extensively studied promiscuous plasmid pMV158 (Lorenzo-Díaz et al., 2014, 2017; López-Aguilar et al., 2015) can form a hexameric ring (RepB_6_). RepB is the only plasmid-encoded RCR initiator with a determined atomic structure (Boer et al., 2009). This protein is composed of two domains: (i) an N-terminal *origin*-binding domain (OBD) (with catalytic tyrosine residue Y^99^), and (ii) a C-terminal oligomerization domain (OD), responsible for formation of the hexameric structure. In this regard, RepB resembles Rep proteins of eukaryotic viruses in which hexameric ring formation determines helicase activity (Boer et al., 2016). However, RepB does not contain a helicase domain. It is believed that the RepB_6_ ring fastens the DNA strand, enabling recruitment of helicase and DNA polymerase. The replisome so formed is highly efficient in duplex unwinding and DNA synthesis, and allows pMV158 to replicate in a broad spectrum of bacterial hosts (Boer et al., 2009). It is not clear how RepB_6_ terminates replication. In one of the proposed models, the flexible structure of RepB_6_ OBDs enables the use the Y^99^ from one monomer for initiation cleavage and the Y^99^ from another monomer for termination cleavage (Boer et al., 2009). OBD flexibility also facilitates *origin* recognition by RepB_6_ (Boer et al., 2016).

The replicons described above have circular genomes. However, non-circular genomes may also replicate using an RC-like mechanism. For example, a variation of RCR named rolling-hairpin replication (RHR) (**Figure 3**) was observed in the adenovirus-associated virus, AAV, a member of the family *Parvoviridae* (Tattersall and Ward, 1976). The genome of this virus is relatively small (∼4700 bp) and comprises only two genes: *rep* – encoding the replication initiator, and *cap* – encoding the capsid formation protein. These genes are located between two inverted terminal repeats (ITRs), whose palindromic sequences promote the formation of characteristic T-shaped structures at both ends of the DNA molecule (**Figure 3**). Interestingly, the AAV *rep* gene may encode up to four peptides, two of which, Rep78 and Rep68, contain motifs that are characteristic of Rep proteins of RCR plasmids.

During infection, the single-stranded DNA of the virus is introduced into a eukaryotic cell and then converted to dsDNA. Synthesis of the complementary strand does not require an RNA primer because DNA replication is initiated from the free 3′-OH end of the ITR acting as a primer (**Figure 3**). However, replication initiated in this way does not lead directly to duplication of the entire AAV genome, and the RHR mechanism must be used to complete the replication round (**Figure 3**). RHR is initiated at a *dso* located within the 3′ ITR. Similarly to the double-stranded *origin* of RCR plasmids and bacteriophages, the *dso* of AAV also comprises two conserved regions: a binding site for the replication protein (RBS) and a DNA cleavage site, known as the terminal resolution site (TRS) (Balakrishnan and Jayandharan, 2014).

### RCR in Conjugal Transfer

Many plasmids and ICEs may be transmitted to other bacterial cells by conjugal transfer (Smillie et al., 2010; Carraro and Burrus, 2014; Cabezón et al., 2015). This process requires the involvement of two gene modules responsible for (i) mating pair formation (MPF) and (ii) DNA transfer and replication (DTR). In the case of elements containing only the DTR module (mobilizable plasmids and integrative and mobilizable elements, IMEs), conjugal transfer is possible only when a compatible MPF module is present *in trans*.

The MPF region usually comprises genes encoding the type 4 secretion system (T4SS) involved in DNA transport and biogenesis of the pilus connecting bacterial conjugation pairs (Garcillán-Barcia et al., 2009; Guglielmini et al., 2014). The DTR region encodes the Mob protein, a relaxase that cleaves one strand of DNA within a specific site known as the *origin* of transfer (*oriT*). The *oriT* region comprises functional *nick* and *bind* sites, which are analogous to those of the *dso* RCR plasmids. Moreover, it has been demonstrated that cleaving one DNA strand in *oriT* also initiates RCR.

The key role of RCR in conjugal transfer is to separate the replication of the two strands of DNA so that it occurs simultaneously in different bacterial cells. The leading strand (+) is replicated in the donor cell and the lagging strand (-) in the recipient cell. This process is more complex than the vegetative plasmid replication described above.

**FIGURE 2:**
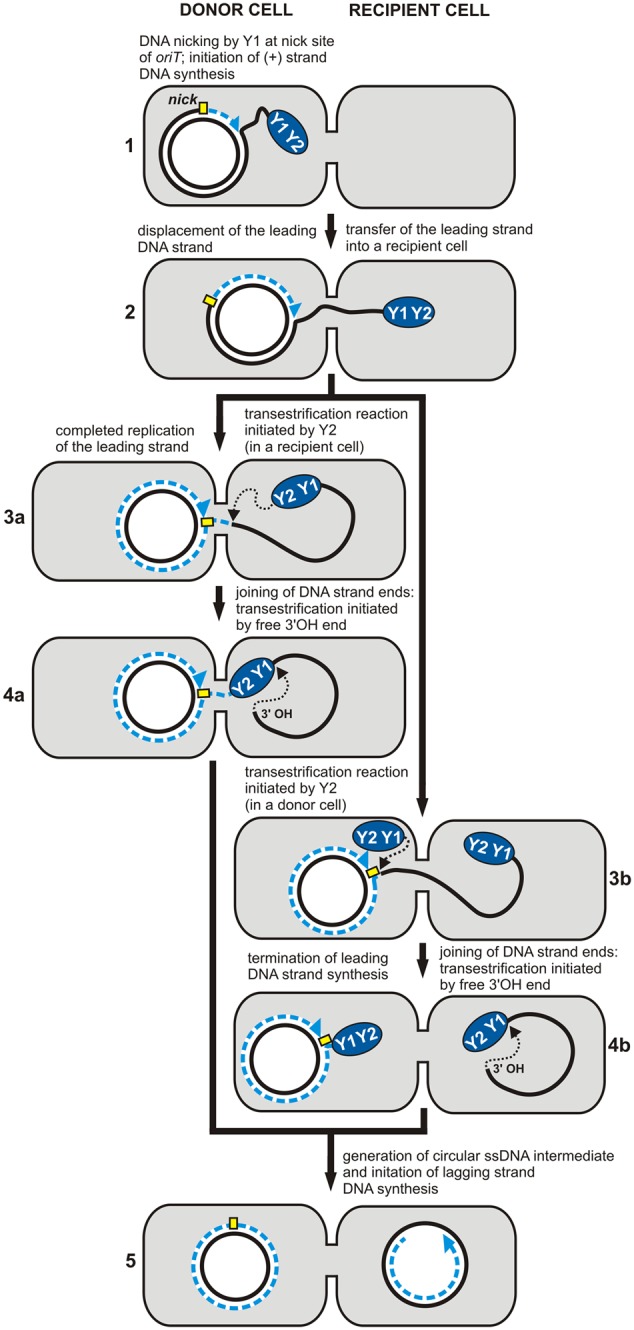
Rolling-circle replication (RCR) in conjugal transfer. The individual stages of DNA synthesis during plasmid conjugal transfer (1–5). The RCR initiation protein (relaxase) containing two conserved tyrosine residues, Y1 and Y2, initiates (+) strand replication at the *nick* site (marked by a yellow rectangle). Both Y1 and Y2 may be involved in DNA processing during the transfer (plasmid R388) (3a, 4a), or only one of them – Y1 (plasmid F) (3b, 4b) (see text for details). Dashed blue lines indicate the nascent DNA strand and blue arrows show the direction of synthesis. Black dotted arrows indicate nucleophilic attack of the catalytic tyrosine residue on the phosphodiester bond in the DNA or of the free 3′-OH DNA end on the phosphotyrosine bond between the enzyme and the DNA (adopted from Chandler et al., 2013).

In conjugal transfer, the relaxase protein (Mob) acts like the Rep protein of plasmids and viruses during RC vegetative replication. The Mob protein also contains tyrosine residues in its active site, which determine the catalytic functions. For example, the TrwC relaxase of the plasmid R388 contains two catalytic tyrosines (Y^18^ and Y^26^) that are essential for DNA transfer (marked Y1 and Y2 in **Figure 2**). According to the model proposed by Gonzalez-Perez et al. (2007), the first tyrosine (Y^18^) is responsible for the initiation of replication at *oriT* in the donor cell, while the second residue (Y^26^) promotes termination of leading strand replication in the recipient cell. The relaxase TraI of the plasmid F also contains two conserved tyrosine residues within its catalytic domain, although only one (Y^16^) is active in DNA processing (marked Y1 in **Figure 2**). This suggests the involvement of two protein molecules acting independently at the initiation and termination sites of the lagging strand (Gonzalez-Perez et al., 2007).

After the initiation of replication, relaxase remains covalently bound to the 5′ end of *oriT*, while the free 3′-OH end is used as a primer for the synthesis of the complementary strand in the donor cell. DNA relaxation induces the formation of a nucleoprotein complex called the relaxosome, which is then targeted via the T4 coupling protein (T4CP) to the components of the T4SS. As a result, the leading DNA strand, along with the relaxosome, is transferred to the recipient cell through the pores formed in the membrane by the T4SS. The single-stranded DNA transferred from the host is recognized by the replication engine of the recipient strain, and it serves as the template for the synthesis of the complementary strand. This completes the round of replication.

Interestingly, some relaxases have additional domains responsible for primase activity. Such multifunctional proteins are transferred to the recipient cell along with the relaxosome complex, where they are involved in the RNA primer synthesis. As a result, the transferred ssDNA is converted to dsDNA even in strains where the lagging strand *origin* is not recognized by the replication engine of the recipient. It is thought that this primase activity of the Mob protein might extend the host range of the MOB module (Henderson and Meyer, 1999).

### RCR in Transposition

Transposable elements (TEs) are the largest group of mobile genetic elements found in bacteria. Usually they are integrated within the genome of the host, but they may change their location by recombination, known as transposition. The transposition is catalyzed by a transposase enzyme encoded by TEs.

**FIGURE 3:**
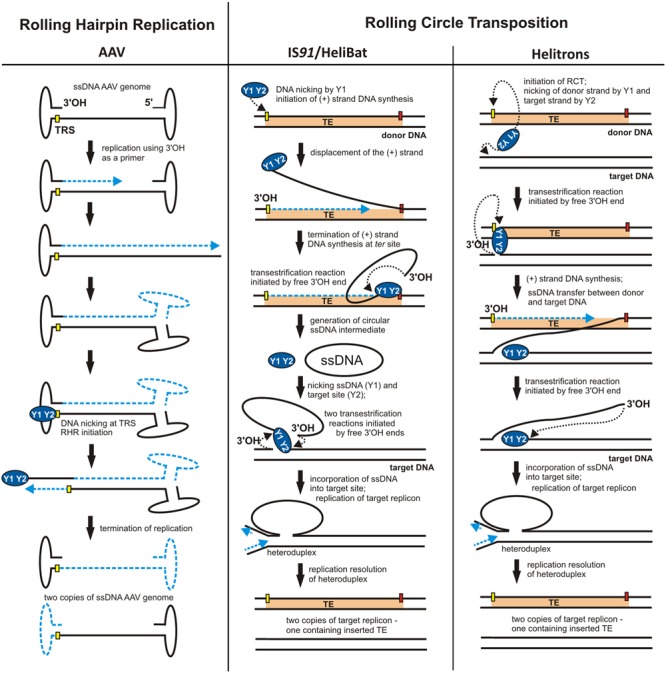
Different applications of RCR. Models for rolling-hairpin replication (RHR) of adeno-associated virus (AAV) and rolling-circle transposition (RCT) of IS*91*/HeliBat and Helitrons. The individual stages of replication are described in the Figure and details are given in the text. ITRs – inverted terminal repeats of AAV. The *nick* sites of the origin are shown as yellow rectangles and the termini of RCT are marked by red rectangles. Dashed blue lines indicate nascent DNA strands and blue arrows show the direction of DNA synthesis. Black dotted arrows indicate nucleophilic attack of the catalytic tyrosine residue on the phosphodiester bond in the DNA or of the free 3′-OH DNA end on the phosphotyrosine bond between the enzyme and the DNA (adopted from Chandler et al., 2013; Thomas and Pritham, 2015; Grabundzija et al., 2016).

A characteristic feature of the majority of TEs is the presence of inverted repeats (IRs) at their termini. These sequences are responsible for interaction with the transposase. During transposition, most TEs also generate direct repeats (DRs) in the target DNA. Among the exceptions to this general model is the insertion sequence IS*91* identified in a hemolytic *Escherichia coli* strain (Zabala et al., 1982). This TE does not contain IR sequences nor does it generate DRs during transposition. Moreover, the amino acid sequence of the IS*91*-encoded transposase (TnpA) does not resemble those of other TE transposases (Mendiola et al., 1992), but it contains motifs typical for RCR initiation proteins, including two strongly conserved tyrosine residues (Y^249^ and Y^253^). Mutations in Y^249^ and Y^253^ result in the loss of TnpA *in vivo* activity (Mendiola and de la Cruz, 1992). Therefore, TnpA is phylogenetically closer to the RCR initiator proteins of plasmids and bacteriophages than to typical TE transposases. These similarities are reflected in the transposition of IS*91* which follows the RCR model (**Figure 3**).

The IS*91* transposition model, called rolling-circle transposition (RCT), involves excision of only one IS*91* strand from the donor site and its integration within the target DNA (sequential cut-out, copy-in mechanism) (Mendiola et al., 1994; Curcio and Derbyshire, 2003). The terminal regions of IS*91* that initiate and terminate the first transposition step are named *ori91* and *ter91*, respectively. TnpA protein cleaves one strand of DNA within *ori91*, which initiates replication of the leading IS*91* DNA strand using the 3′-OH terminus as a primer (**Figure 3**). The displaced DNA strand is cleaved by TnpA at *ter91* and converted into a RCR-specific circular ssDNA intermediate (Garcillán-Barcia et al., 2001). In the second step, transposase catalyzes the incorporation of the single-stranded intermediate into the new target site. There, following synthesis of the complementary strand (during target DNA replication), a complete copy of IS*91* is reproduced (**Figure 3**) (Curcio and Derbyshire, 2003).

IS*91* is the archetype of the IS*91* family of insertion sequences, whose representatives, also capable of RCT, are found in the genomes of bacteria belonging to various taxonomic groups. Interestingly, other TEs, unrelated to IS*91*, but following the RCT model, have been identified in plant and animal genomes. These elements, known as helitrons, belong to the 2nd class of eukaryotic transposons, first detected by *in silico* analysis of genomic sequences. In some cases, these elements represent as much as 6% of the genetic material of the host. They are particularly abundant in the genomes of *Heliconius melpomene* (butterfly), *Myotis lucifugus* (bat) and *Zea mays* (corn).

Helitrons, like members of the IS*91* family, do not generate DR sequences during transposition and their transposases resemble RCR initiator proteins (Thomas and Pritham, 2015). Moreover, ssDNA intermediates were identified for one of the helitrons of *M. lucifugus* (HeliBat), suggesting that its transposition mechanism is analogous to that of IS*91* (Grabundzija et al., 2016). For other helitrons, a different RCT mechanism is postulated where the transposase simultaneously cleaves single DNA strands within the donor and target sequences, which is consistent with the copy-in model. The DNA strand removed during replication is then directly transposed to the target site without generating ssDNA intermediates (**Figure 3**) (Curcio and Derbyshire, 2003; Thomas and Pritham, 2015).

Analysis of the RCT mechanism has shown that in many cases RC transposases do not recognize the termination sites located at the end of each TE. This may result in the transposition of genomic DNA fragments located in the immediate vicinity of the TE. Such a phenomenon is frequently observed in the case of helitrons and ISCRs (insertion sequence common regions) (Toleman et al., 2006). Moreover, a correlation between the occurrence of IS*91* family members and the proliferation of virulence genes in bacteria was reported (Garcillán-Barcia and de la Cruz, 2002). This indicates the importance of RCT in shaping genomes and its role in the dissemination of non-viable genetic information *via* HGT (Thomas and Pritham, 2015).

## Characteristics of RCR Initiator Proteins

As explained above, RCR initiator proteins break the continuity of one DNA strand at the *nick* site. This is a multi-step transesterification reaction (**Figure 1B**) which is reversible and does not require ATP (Byrd and Matson, 1997). To date, two main phylogenetically distinct families of initiator proteins with endonuclease activity have been described: HUH and Rep_*trans*. Although the mechanism of these endonucleases is very similar, crystallographic studies have revealed significant differences in the structure of their catalytic domains (Chandler et al., 2013; Carr et al., 2016).

### HUH Endonucleases

Endonucleases containing the HUH motif (H-H – conserved histidine residues, U – hydrophobic amino acid) constitute a diverse group of proteins. They are involved in various processes including (i) the initiation of replication of bacterial plasmids (Rep), bacteriophages and eukaryotic viruses, (ii) conjugal transfer of DNA (Mob), and (iii) transposition of TEs (Tnp).

The RCR Rep proteins of plasmids are divided into two families: (i) Rep_*1* (PF01446), comprising the pC194 and pUB110 initiator proteins, and (ii) Rep_*2* (PF01719), the pMV158 and pE194 Rep proteins (Punta et al., 2012). HUH endonucleases from viruses are more diverse. They are classified into four families: (i) Phage_*GPA* (PF05840), whose prototype is the phage ΦX174 protein A; (ii) Viral_*Rep* (PF02407), which includes eukaryotic replication proteins, e.g., circovirus PCV2; (iii) Gemini_*AL1* (PF00799), responsible for the replication of geminiviruses; and (iv) Rep_*N* (PF08724), comprising proteins that initiate the replication of linear parvovirus genomes, including AAV (Punta et al., 2012).

The majority of relaxases (Mob proteins) encoded in the DTR regions of plasmid transfer systems, ICE and IME, are also HUH endonucleases. These proteins are classified into six families (Garcillán-Barcia et al., 2009), four of which (MOB_F_, MOB_Q_, MOB_P_ and MOB_V_) group enzymes with catalytic domains typical of HUH endonucleases. The two remaining families, MOB_H_ and MOB_C_, are significantly different. Although MOB_H_ proteins share some similarities with the HUH enzymes at the amino acid sequence level (e.g., the presence of a three-histidine HHH motif), there are no crystallographic data to confirm their structural similarity (Garcillán-Barcia et al., 2009; Chandler et al., 2013). This is not the case for members of the MOB_C_ family, which possess a PD-(D/E)XK-like nuclease domain and lack motifs typical of HUH enzymes. Therefore, they constitute an evolutionarily independent family (Francia et al., 2013).

Apart from their involvement in replication and conjugation, HUH endonucleases can also act as transposases for (i) IS*91*, ISCR and helitrons, (ii) IStrons – mosaic elements combining the features of TEs and introns (Tourasse et al., 2014), and (iii) repetitive extragenetic palindromes (REP) classified as bacterial interspersed mosaic elements (BIMEs) (Ton-Hoang et al., 2012). However, not all transposases containing the HUH motif perform RCT, e.g., those belonging to the IS*200*-IS*608* bacterial transposon family (He et al., 2015).

The activity of all HUH endonucleases is similar. They first cleave DNA at a specific location and covalently bind to the free 5′-end (Chandler et al., 2013). This produces a stable nucleoprotein complex, and the released 3′-OH end is then used as a replication primer. Three characteristic motifs present in the catalytic domain of HUH endonucleases determine the activity of these proteins (Ilyina and Koonin, 1992): (i) motif I, responsible for the recognition of the DNA sequence; (ii) motif II, with two histidine residues, responsible for the binding of divalent cations (Mg^2+^ or Mn^2+^) (metal ions are directly involved in catalysis, plus they participate in catalytic domain stabilization and *origin* recognition), (iii) motif III, containing a catalytic tyrosine residue that carries out a nucleophilic attack on the phosphodiester bond (Yang, 2008; Lorenzo-Díaz et al., 2011; Chandler et al., 2013; Ruiz-Masó et al., 2016). Modified forms of these motifs occur in several HUH endonucleases (Koonin and Ilyina, 1992). For example, HUH proteins may contain either one or two catalytic tyrosine residues in their active site, and can thus be divided into two groups: 1Y and 2Y (Chandler et al., 2013). There may also be variations in the order of the conserved motifs within the catalytic domain (Ilyina and Koonin, 1992). For example, plasmid Rep proteins have motifs arranged in the order I-II-III, while in Mob proteins the scheme is reversed (III-II-I). This difference, however, is insignificant at the level of the tertiary protein structure because the active sites of proteins from both groups are similar (Dyda and Hickman, 2003).

### Rep_*trans* Endonucleases (PF02486)

The Rep_*trans* family comprises proteins involved in the initiation of (i) replication of multiple bacterial plasmids, e.g., pT181, pC221 and pUB112 (Projan and Novick, 1988; Balson and Shaw, 1990), (ii) replication of bacteriophages, including CTXΦ (Waldor and Mekalanos, 1996), and (iii) conjugal transfer of some integrative elements, such as ICE*Bs1* (Lee and Grossman, 2007) and Tn*916* (Rocco and Churchward, 2006). Recently, the Rep_*trans* proteins have been classified within the MOB_T_ family (Punta et al., 2012), even though they do not share significant similarity with HUH endonucleases (homology of ≤10%) and they lack conserved domains characteristic of HUH proteins (Carr et al., 2016). However, Rep_*trans* and HUH proteins have some common features, as they both (i) use tyrosine as a nucleophile, (ii) catalyze a transesterification reaction resulting in the formation of a phosphotyrosine bond between the protein and the 5’-end of the DNA, and (iii) are dependent on divalent cations (Koepsel et al., 1985; Dempsey et al., 1992; Zhao and Khan, 1997; Khan, 2003; Pastrana et al., 2016).

Until recently there were no crystallographic data available for Rep_*trans* family proteins. The first three-dimensional structures of the main domain of the Rep protein of plasmid pSTK1 from *Geobacillus stearothermophilus* and fusion proteins comprised of domains of *Staphylococcus* spp. Rep proteins were determined by Carr et al. (2016), and they confirmed the difference between the Rep_*trans* and HUH proteins. The catalytic tyrosine residues of Rep_*trans* proteins are located within a β-sheet structure, whereas the YxxK/H motif of HUH endonucleases is always part of an α-helix. Crystallographic analysis also revealed that the binding of divalent cations (Mg^2+^ or Mn^2+^) by Rep_*trans* proteins is mediated by an arrangement of three acidic amino acids – aspartate (D), aspartate (D) and glutamate (E) (Carr et al., 2016). Moreover, the active sites of the analyzed Rep_*trans* proteins have an almost identical structure consisting of five regions: (i) motif N’, containing a highly conserved aspartic acid residue (**D**), (ii) motif I – Rx**D**, (iii) motif II – T/SxY/ExG, (iv) motif III – **Y**xKxxE, and (v) motif IV – W/LxRx**E** (the DDE motif residues and the catalytic tyrosine Y are marked in bold). Similar motifs are also present in the replication proteins of bacteriophages M13 and Cri, representing the Phage_*CRI* family (PF05144). This may indicate their close relationship to the Rep_*trans* family of proteins (Carr et al., 2016).

## The Multifunctional Character of RCR-Initiating Enzymes

**FIGURE 4:**
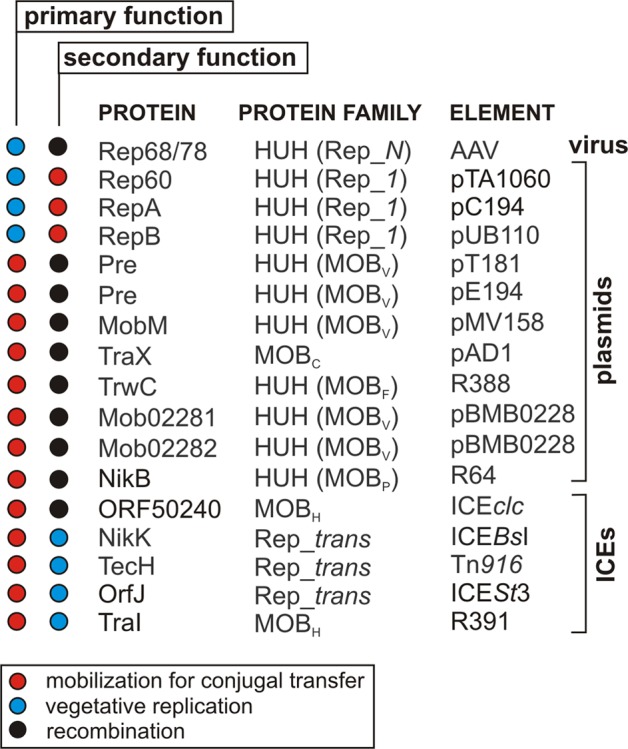
Multifunctional RCR initiator proteins encoded by different types of genetic elements.

RCR initiators are encoded in the genomes of many bacteria, eukaryotic MGEs and viruses. For a long time it was thought that except for the bifunctional Mob/Pre proteins (described below), these enzymes were specialized in performing dedicated biological processes, and so were named Rep, Mob or Tnp proteins. Surprisingly, recent reports have demonstrated the multifunctionality of these RCR initiators (e.g., Wang et al., 2013; Wright and Grossman, 2016; González-Prieto et al., 2017). It is now evident that these proteins, depending on their mechanism of action, can conduct different biological processes associated with RCR (**Figure 4**).

### Proteins Responsible for Mobilization for Conjugation Transfer (Mob) Also Act as Recombinases/Integrases (Int)

In the 1980s, it was noted that some HUH endonucleases may initiate two independent processes. These included the plasmid recombination (Pre) proteins of three Gram-positive bacterial plasmids: pT181 and pE194 of *Staphylococcus aureus*, and pMV158 of *Streptococcus agalactiae* (Gennaro et al., 1987). These plasmids have the ability to form co-integrates within a strictly defined RSA (recombination site A) sequence. The formation of such structures is independent of the RecA protein, which usually plays a major role in homologous recombination, but requires the presence of the plasmid-encoded Pre protein (Gennaro et al., 1987). Further studies revealed that Pre proteins initiate the transduction of pT181, pE194 and pMV158 (Priebe and Lacks, 1989). Since these proteins can act as both relaxases (Mob) and recombinases they were initially classified within the Mob/Pre family, but they are now included in the MOB_V_ family (Garcillán-Barcia et al., 2009).

Pre proteins are not an isolated example of multifunctional endonucleases. Similar properties were reported for several other distantly related relaxases, including TraX from the plasmid pAD1 (MOB_C_ family) (Francia and Clewell, 2002), NikB (MOB_P_) from the plasmid R64 (Furuya and Komano, 2003), TrwC from the plasmid R388 (MOB_F_ family) (César et al., 2006) and a relaxase from an ICE*clc* element (MOB_H_ family) (Miyazaki and van der Meer, 2011; Bellanger et al., 2014). However, not all relaxases can act as recombinases despite their similarity, e.g., the TraI proteins from plasmids F and pKM101, which are related to TrwC (MOB_F_ family). It may be concluded that the recombinase activity of some relaxases is not dependent on the catalytic properties of these enzymes (César et al., 2006).

The factors that actually determine the recombinogenic properties of relaxases have yet to be identified. However, it has been shown that in some cases such activity may be stimulated by other proteins, e.g., NikA from plasmid R64 (Furuya and Komano, 2003) or TrwA from R388 (Agúndez et al., 2012). The structure of the *oriT* itself and the genetic context in which it occurs are also important. This was demonstrated by studies of the ICE*clc* model, which contains two *origins* of transfer (*oriT1* and *oriT2*) recognized by the same relaxase. In this case, relaxation-dependent recombination exclusively involves the *oriT1* sequence, despite the fact that both *origins* are fully functional and can be used interchangeably during the initiation of conjugal transfer (Miyazaki and van der Meer, 2011).

Relaxation-catalyzed recombination may also lead to the formation of fusion replicons composed of various co-occurring plasmids. This was observed for the plasmid pBMB0228 of *Bacillus thuringiensis*, a cointegrate of two RCR replicons bearing related DTR modules. Each of the Mob proteins encoded by these modules (Mob02281 and Mob02282, 86% amino acid sequence identity) may interact with both *oriT* regions and can catalyze the formation or separation of co-integrates (Wang et al., 2013). These recombination events occur within a short (24 bp) sequence shared by both *oriT*s, containing nearly identical *nick* sites (Wang et al., 2013).

The generation of transient fusion replicons by site-specific recombination has significant implications for the evolution of plasmids. Replicons with a mosaic structure and unique properties (e.g., increased host range or conjugal transferability) might be created in this way.

Relaxase-catalyzed recombination can also occur during conjugal transfer. For example, it was demonstrated that the TrwC protein (HUH endonuclease) is transported to the recipient cell, where it catalyzes the integration of the transferred plasmid within the genome of the new host. Integration occurs only when the target sequence *oriT* R388 is present within the chromosome or a plasmid already residing in the recipient cell. More intriguingly, *oriT* R388-like sequences were also identified in the human genome (on chromosomes 5 and X) and these sequences could be the target for TrwC-catalyzed integration (Agúndez et al., 2012). Therefore, it might be possible to use TrwC relaxase to construct vectors that are potentially useful in gene therapy (González-Prieto et al., 2013, 2017).

### Proteins Initiating Replication of Viral Genomes (Rep) Also Promote Their Chromosomal Integration (Int)

The *rep* gene of the previously mentioned adenovirus-associated virus can encode four Rep proteins formed by alternative splicing of transcripts and the activity of two internal promoters. The two largest proteins, Rep78 and Rep68, participate in the RHR initiation of the autonomous form of the AAV genome. In this case, the presence of an adenovirus in the cell is necessary for AAV replication. In the absence of virus, the AAV genome is integrated into the host chromosome and the lysogenic cycle is initiated. AAV is the only known eukaryotic virus that preferentially integrates at a strictly defined site, AAVS1 (adeno-associated virus integration site 1), located within chromosome 19 of the human genome (Smith, 2008). Because of the similarity between the AAVS1 sequence and the RBS and TRS regions of AAV, this virus has become a convenient model for gene therapy vectors (Balakrishnan and Jayandharan, 2014). However, results obtained using high-throughput DNA sequencing have shown that only 45% of AAV insertions occur within AAVS1. The other sites of integration, apart from being similar to the RBS sequence of the virus, were characterized by high transcriptional activity (Janovitz et al., 2013).

The incorporation of the wild-type viral genome into the host cell chromosome depends on the activity of the Rep78 and Rep68 proteins (Rep78/68) (Balagúe et al., 1997; Surosky et al., 1997). However, it was found that even a mutant virus, in which the DNA is not processed by Rep78/68, can integrate into the genome of the host with comparable efficiency (Young and Samulski, 2001). Further studies showed that integration of the virus occurs not at the position where the Rep78/68 nicks the DNA, but at some distance from this cleavage site. In addition, several copies of the viral genome are often integrated at the same target site in the form of head-to-tail concatamers.

Interestingly, recombination events within the target sequence may occur in the absence of AAV DNA. In such cases, the only requirement is the presence of Rep78/68 protein in the human cell (Young et al., 2000). The mechanism of this process is different from reactions catalyzed by previously characterized integrases. According to the model proposed by Young and Samulski (2001), Rep78/68 initiates local RCR replication by nicking DNA at the TRS in the target sequence. Successive initiations lead to the formation of single-stranded DNA intermediates with Rep attached to the 5′-ends, which are the “hot spots” for recombination events. It is postulated that Rep78/68 recruits cellular proteins involved in DNA recombination, including components of the non-homologous end joining system (NHEJ) (Daya et al., 2009). This system, found in both prokaryotic and eukaryotic cells, is responsible for the repair of double-stranded breaks in DNA (Nash et al., 2009). According to an alternative model, the Rep78 and Rep68 proteins of AAV are contacted by two DNA molecules (viral genome and host chromosome) while interacting with AAVS1 and the viral DNA. This results in the transition of DNA polymerase from the chromosome template to the AAV DNA template and, consequently, in the integration of the viral genome (Henckaerts et al., 2009; Krupovic and Forterre, 2015).

### Proteins Initiating ICE Conjugal Transfer (Mob) Are Also Involved in Vegetative Replication (Rep)

Integrative and conjugative elements are a heterogeneous group of MGEs (Guglielmini et al., 2011). They are characterized by the presence of (i) a recombination system encoding an integrase responsible for ICE integration-excision, and (ii) a conjugal transfer system (Carraro and Burrus, 2014).

Integrative and conjugative elements usually occur in the chromosome-integrated form. They may, however, be excised from the genome and form autonomous circular forms (dsDNA) that mimic plasmids, although they lack their own replication systems. Such circular forms of ICE may be reintegrated into the host genome or transferred via conjugal transfer to another bacterial cell, where they integrate within the recipient genome. These elements combine the features of (i) transposons (ability to integrate within DNA, changing location within a host genome), (ii) plasmids (circularity, coding for their own conjugal transfer system), and (iii) bacteriophages (recombination systems involved in DNA integration and excision).

ICE*Bs1* (20.5 kb) from a strain of *Bacillus subtilis* is one of the model elements in ICE biology research (Auchtung et al., 2016). Surprising results have been reported indicating that the free circular form of ICE*Bs1*, despite the absence of a replication system, can replicate autonomously (Lee et al., 2009; Grohmann, 2010). It was also shown that the replication of this ICE is initiated within the *oriT* by the ICE*Bs1* relaxase NicK, which initiates both conjugal transfer and vegetative replication of this element. Thus, the dogma that DNA replication initiated within DTR modules occurs only during conjugal transfer was disproved.

Autonomous vegetative replication depending on *oriT* and relaxase has also been demonstrated for other ICEs, e.g., Tn*916* (18 kb) the archetype of the Tn*916*/Tn*1545* family (Wright and Grossman, 2016), ICESt3 (28 kb) a representative of the ICE*St*1/ICE*St*3 family (Carraro et al., 2016), and R391 an 89-kb element from the STX/R391 family (Carraro and Burrus, 2014). The STX/R391 family is comprised of very large ICEs (sometimes > 100 kb) isolated from *Gammaproteobacteria* (Carraro and Burrus, 2014). The vegetative RC replication of such large DNA molecules has not been previously observed. It was believed that this replication mechanism was specific only for small (up to ∼10 kb) high copy-number replicons (Khan, 1997; Ruiz-Masó et al., 2015). It should be emphasized that the R391 relaxase (TraI) is not phylogenetically related to analogous proteins encoded by elements from other ICE families, and therefore it was classified to a distinct family of relaxases – MOB_H_ (Garcillán-Barcia et al., 2009). It is noteworthy that the autonomous replication of ICE circular forms is not limited to elements encoding Rep_*trans* relaxases, as was once thought, and it undoubtedly plays an important role in ICE biology.

If the ICE does not excise from the host genome its maintenance in daughter cells will be guaranteed. However, following excision of an ICE from the host genome, replication is critical for its maintenance as a single copy in an autonomous form. Without replication, every cell division could result in the formation of segregated ICE-free clones. Duplication of ICE circular forms by RCR enables a copy of the element to be passed to the daughter cells, which stabilizes their presence in the bacterial population.

### Proteins Initiating the Replication of Plasmids (Rep) Also Initiate Conjugal Transfer (Mob)

Analysis of the ICE*Bs1* conjugation element from *Bacillus subtilis* led to the observation that it is capable of mobilizing various RC replicons including pC194, pBS42 (bearing the REP module from pUB110) and pHP13 (with a pTA1060-like REP). Interestingly, these replicons do not carry DTR systems characteristic of other mobilizable plasmids, and thus lack relaxase genes and *oriT* regions. A more detailed analysis failed to demonstrate a role for the ICE*Bs1* NicK relaxase in plasmid mobilization. Instead it was shown that the vegetative RCR-initiating proteins (Rep) of these plasmids can act as relaxases (Mob) involved in conjugal transfer. To initiate transfer, such Rep proteins cleave the DNA at the *nick dso*, which then serves as an *oriT* (Lee et al., 2012). The coupling protein ConQ encoded by ICE*Bs1* plays an important role in the transfer, as it probably binds to the DNA-Rep/Mob relaxosome and directs this complex to the components of the T4SS. As part of the ICE*Bs1* transfer system the ConQ protein interacts with the NicK parental relaxase, a member of the Rep_*trans* endonuclease family, as well as the pC194, pUB110 and pTA1060 Rep endonucleases belonging to the HUH Rep*_1* family. This is particularly noteworthy since the Rep proteins of pC194, pUB110 and pTA1060 are not phylogenetically related to NicK, and there are no characteristic translocation signals (TSs) in their sequences enabling relaxosome transfer via the T4SS machinery (Lang et al., 2010; Alperi et al., 2013). The molecular basis of these interactions has yet to be determined, and the function of the Rep proteins of pC194, pUB110 and pTA1060 in plasmid conjugal transfer remains uncharacterized.

The aforementioned results raise doubts concerning the validity of plasmid classification based on their mobilization potential, if this characteristic is strictly determined by the presence of DTR or *oriT* systems. According to these criteria the plasmids pC194, pBS42 and pHP13 are non-mobilizable, which is refuted by the experimental data. Most previously analyzed plasmids do not carry DTR systems. However, if a DTR system is not always necessary for conjugal transfer, these plasmids might be mobilizable and play a larger role in HGT than was previously thought. The demonstration of a dual role for Rep/Mob proteins, being capable of initiating vegetative and conjugative RCRs, sheds new light on the biology of the RCR plasmid group as well as ICE elements commonly found in many bacterial genomes (Lee et al., 2012).

## Conclusion

Rolling-circle replication is involved in various processes conducted by genetic elements, including (i) replication of their autonomous forms (plasmids, bacteriophages, viruses and ICEs), (ii) conjugal transfer (plasmids, ICEs), (iii) transposition (TEs), and (iv) recombination. It should be noted that parts of the host chromosome may also be multiplied in a RC manner. Such replication is mediated by helitrons and ISCRs (able to mobilize adjacent chromosomal genes for transposition) as well as by some conjugative plasmids, which temporarily integrate into the host chromosome. In the latter scenario (as shown in the case of plasmid F), conjugation initiated by the integrated plasmid may result in replication and transfer of a large part of the host chromosome to a recipient cell (Griffiths et al., 1999).

In this review, we have summarized various RCR models developed for individual MGE groups. We have paid particular attention to the multifunctional nature of the RCR initiator proteins, which has not been comprehensively described before. These versatile proteins can determine the course of different biological processes based on RCR. The examples of Mob and Rep proteins that we have discussed show integrase (Int) activity. Other Mob proteins are capable of initiating vegetative RC replication (Rep activity) and some Rep proteins can mobilize plasmid DNA for conjugal transfer (Mob activity) (**Figure 4**). There are currently no empirical data concerning multifunctional proteins representing the third group of RCR initiators – the Tnp proteins. However, only single examples of this group have been characterized in detail and most of these proteins were analyzed using only bioinformatics tools.

It appears that two factors are critical for the multifunctional activity of RCR initiators: (i) the universal character of the DNA breaking/joining reaction they catalyze, and (ii) the genetic context in which the RCR initiator is present. The type of protein seems to be less important since HUH, Rep_*trans* as well as MOB_C_ and MOB_H_ endonucleases can all act as multifunctional proteins.

It may be concluded that many MGEs possess RCR genetic modules that can have additional features, which remain to be discovered, and the eventual characterization of these features may lead to the overturning of widely accepted dogma. For example, plasmids and viral genomes are no longer considered to be the only genetic elements capable of duplicating their genomes by autonomous replication. Recent studies have shown that integrative and conjugal elements should now be included in this group because their DTR modules also initiate vegetative RC replication. Moreover, this ability is likely to be a characteristic of ICEs (Burrus, 2017), which redefines the properties of this group of MGEs.

Further discoveries in this field may lead to the reclassification of some plasmids. Until now, two main plasmid groups have been distinguished: mobilizable (containing a DTR module, auxiliary transfer system-dependent), and non-mobilizable (i.e., unable to transfer). However, since some Rep proteins were shown to be involved in mobilization, a new subgroup of mobilizable plasmids, the RCR plasmids without DTR modules, should be created. The above examples highlight the need for detailed functional characterization of a wider range of MGEs containing RCR modules.

Research on RCR has a long history. Nonetheless there are still many unanswered questions about this model of DNA replication, as well as the nature and diversity of proteins involved in the replication process. The vast majority of identified RCR initiators are classified within two relatively well-characterized families, HUH and Rep_*trans*. Little is known about the molecular characteristics of other RCR initiators belonging to the MOB_C_ and MOB_H_ families.

Current research on RCR is mainly focused on (i) mechanistic studies involving advanced biophysical methods, (ii) determination of the three-dimensional structures of RCR initiators, and (iii) involvement of host factors in this process (Ruiz-Masó et al., 2015; Pastrana et al., 2016). This review highlights the need for in-depth studies on the multifunctional activity of RCR initiators in order to understand the molecular basis of this phenomenon. It will be of particular interest to define the specific factors and conditions responsible for the switch between the distinct biological functions of initiator proteins. It is equally important to identify how common this phenomenon is among different types of MGEs. Thus further studies are required to fill the many remaining gaps in our knowledge concerning the process of RCR – one of the main driving forces of HGT.

## Author Contributions

PW and DB designed the study. PW wrote the draft manuscript. DB and GP discussed, revised and modified the manuscript to its final version. All authors approved the manuscript for publication.

## Conflict of Interest Statement

The authors declare that the research was conducted in the absence of any commercial or financial relationships that could be construed as a potential conflict of interest.
